# Palestinian Patients with Cancer at King Hussein Cancer Center

**DOI:** 10.3389/fonc.2022.997492

**Published:** 2022-09-30

**Authors:** Razan Mansour, Justin Z. Amarin, Abdallah Al-Ani, Maysa Al-Hussaini, Asem Mansour

**Affiliations:** ^1^ Office of Scientific Affairs and Research, King Hussein Cancer Center, Amman, Jordan; ^2^ Department of Pathology and Laboratory Medicine, King Hussein Cancer Center, Amman, Jordan; ^3^ Department of Radiology, King Hussein Cancer Center, Amman, Jordan

**Keywords:** Cancer care facilities, Gaza Strip, Oncology service, Palestinians, West Bank

## Abstract

**Background:**

The Palestinian Ministry of Health (MoH) routinely refers Palestinian patients with cancer to King Hussein Cancer Center (KHCC), the largest cancer center in the Middle East.

**Aims:**

We aimed to describe the characteristics of Palestinian patients with cancer.

**Methods:**

We performed a retrospective chart review of all Palestinian patients with cancer who were treated at KHCC during 2018 and 2019, of which demographic and clinical characteristics were presented.

**Results:**

We initially started with 521 cases, out of which 41 (7.9%) cases were excluded due to misdiagnosis as malignant on pathology review. We included 480 patients with a confirmed diagnosed of cancer. Most patients were adults (88.8%) with a mean age of 50.0 ± 15.0 years ranging from 19 to 87 years. The most common cancer sites in adult men, who comprised 46.9% of the cohort were the hematolymphoid system (23.5%), followed by the digestive system (17.5%), and lung and pleura (11.5%). In women (53.1%), the most common cancer sites were the breast (46.0%), followed by the digestive system (15.0%), and the hematolymphoid system (10.2%). Children and adolescents accounted for 11.3% of the total cases, among which the hematolymphoid system was the most common cancer site (50%), followed by the brain (14.8%). About 36.0% of all patients presented with advanced-stage disease (i.e., distant metastasis).

**Conclusion:**

The most common cancer sites in our cohort are generally similar to data from the Palestinian territories. Many patients presented with advanced-stage disease, which signals the need for awareness campaigns and screening programs. Benign tumors are misdiagnosed in many patients as cancer. The limited resources and facilities including human resources remain important challenges to the proper and timely diagnosis and management of cancer among Palestinians living in the Palestinian Territories.

## 1 Introduction

Since its inception, the Palestinian Authority (PA) has strived to provide Palestinians with better access to health care services (1). However, limitations in infrastructure and human resources have prevented the health care system in the Palestinian territories from becoming independent (2–4). In addition, the limitations for private cancer care in the Palestinian territories include a shortage of capable cancer specialists, restricted access to medical technology and equipment, and decreased funding from international organizations such as the World Health Organization and countries such as the United States (5). Health insurance from the Palestinian Ministry of Health (MoH) is the only available coverage for cancer care in the Palestinian territories. The insurance usually covers referrals to hospitals in neighboring countries that have advanced facilities for cancer care, such as King Hussein Cancer Center (KHCC) in Jordan (6). In addition to the MoH, other sources of financial coverage include King Hussein Cancer Foundation (through the Goodwill Fund) and direct cash payments.

Cancer remains one of the leading causes of death in the Middle East and represents a significant challenge to many burdened health care systems (7–10). In the Palestinian territories, cancer is the second leading cause of death (11). According to the latest statistics reported by GLOBOCAN 2020, Gaza Strip and the West Bank hosted a total of 4,779 diagnosed cancer cases in that year (12). The age-standardized incidence rate is 158 (per 100,000 persons), and the age-standardized mortality rate is 103.5 (per 100,000 persons); both of which were higher in males compared to females. These number saw a marked increased from projections made for 2015 (13). The increasing burden of cancer, coupled with a limited health care system, represents an additional health care challenge in the Palestinian territories. The most common types of cancer diagnosed in Palestinian patients are similar to worldwide trends (14). Lung cancer is the most diagnosed type of cancer with an incidence rate of 5.2 cases (per 100,000 persons) in men, followed by leukemia and colorectal cancer. In Palestinian women, breast cancer is the most diagnosed type of cancer, followed by colorectal cancer and leukemias (5).

King Hussein Cancer Center is a comprehensive cancer center in Amman, Jordan that serves adult and pediatric patients from Jordan and other countries in the Middle East and North Africa (15). The center is crucial for the cancer care of Palestinian patients because the Palestinian territories lack a comprehensive cancer center. In this study, we describe the characteristics of all patients referred from the Palestinian MoH for cancer care at KHCC between January 2018 and December 2019. We report the demographic characteristics of the patients, their financial coverage, distribution of cancer types, and most common treatment modalities. We also discuss the challenges faced by Palestinian patients. Finally, we provide recommendations for the management and treatment of cancer in the Palestinian territories.

## 2 Materials and methods

We performed a retrospective chart review of all Palestinian patients with cancer who were treated at KHCC between January 2018 and December 2019, who are residents of the Palestinian territories. We sequentially included all patients registered in the institutional Cancer Registry (established July 2006). We filtered out nonunique records and excluded patients with benign tumors that were initially misdiagnosed as malignant. We retrieved the following data from the registry: date at first contact, age at diagnosis, sex, financial coverage, cancer site, histopathology, treatment, and SEER summary stage. We used IBM SPSS Statistics 23 to perform data analyses. First, we described the demographic and clinical characteristics of the full cohort. We then stratified the cohort according to age, sex, or cancer site, and described the clinical characteristics of each strata. We summarized continuous data as means and standard deviations and categorical data as absolute frequencies and percentages.

**Figure 1:**
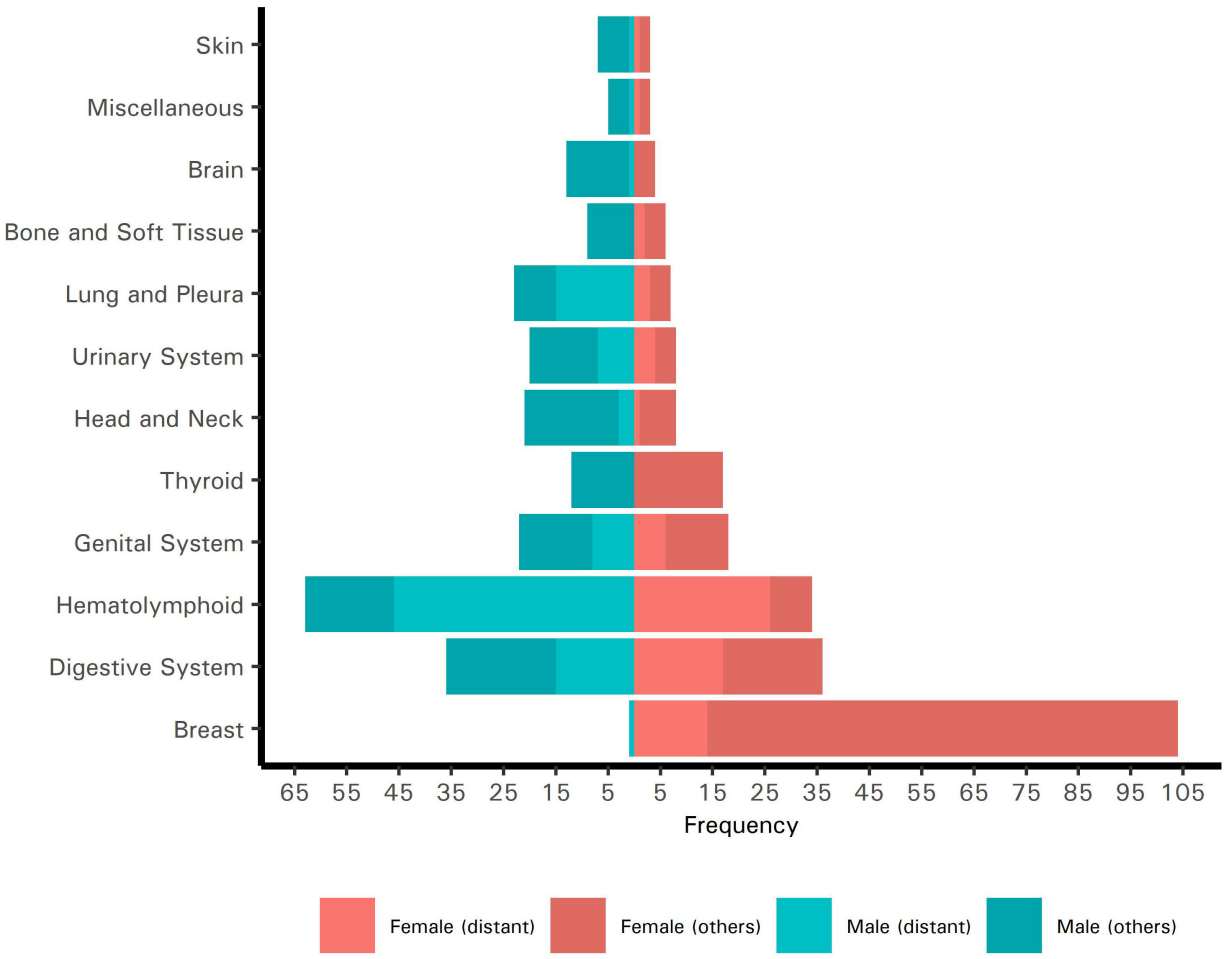
Pyramid plot of the site-specific frequencies of cancers in Palestinian patients who presented to King Hussein Cancer Center in 2018 and 2019 (*N* = 480) stratified by sex. The frequencies of cancers with distant metastases are indicated.

## 3 Results

### 3.1 Demographics

We screened 1,011 records for eligibility, of which 521 records (51.5%) were unique and devoid of any duplicates. Of the 521 patients, we excluded 41 (7.9%) with benign tumors who were previously misdiagnosed. The most common sites of the misdiagnosed tumors were the breast (22.0%), central nervous system (19.5%), pituitary gland (12.2%), digestive system (12.2%), and soft tissues (9.8%). We included, in the final analysis, all remaining 480 patients, 186 (38.8%) and 294 (61.3%) of whom presented to the center in 2018 and 2019, respectively (Refer to **Figure 1**). Of the total, 248 patients were female (51.7%) and 232 (48.3%) were male. The mean age at diagnosis was 45 ± 19 years; 54 patients (11.3%) were children or adolescents (<18 years old) and 426 (88.8%) were adults (≥18 years old). Most patients were financially covered by the Palestinian MoH (n = 435, 90.6%). This cohort of Palestinian patients represented 5.3% of all patients treated at KHCC during 2018 and 2019.

### 3.2 Clinical characteristics

The most common cancer sites, in order, were the breast, hematolymphoid system, and digestive system. **Table 1** describes the site-specific frequencies of cancers among Palestinian patients stratified by age and biological sex. Together, these sites accounted for 274 cancers (57.1%). We describe the SEER summary stage of all cases in **Table 2**. Of all patients, 239 (49.8%) received chemotherapy, 210 (43.8%) underwent surgery, 114 (23.8%) received radiotherapy, 92 (19.2%) received palliative therapy, 39 (8.1%) received hormonal therapy, 16 received immunotherapy (3.3%), and 16 (3.3%) underwent bone marrow transplantation.

**Table 1:** Site-specific frequencies of cancers in Palestinian patients who presented to King Hussein Cancer Center in 2018 and 2019 (N = 480) stratified by age and biological sex.

Site	Total(n = 480)*n* (%)	Female(n = 248)*n* (%)	Male(n = 232)*n* (%)	Adults(n = 426)*n* (%)	Women(n = 200)*n* (%)	Men(n = 226)*n* (%)	Children and adolescents(n = 54)*n* (%)	Girls and female adolescents(n = 22)*n* (%)	Boys and male adolescents(n = 32)*n* (%)
Breast	105 (21.9)	104 (41.9)	1 (0.4)	105 (24.6)	104 (46.0)	1 (0.5)	0 (0.0)	0 (0.0)	0 (0.0)
Hematolymphoid System	97 (20.2)	34 (13.7)	63 (27.2)	70 (16.4)	23 (10.2)	47 (23.5)	27 (50.0)	11 (50.0)	16 (50.0)
Digestive System	72 (15.0)	36 (14.5)	36 (15.5)	69 (16.2)	34 (15.0)	35 (17.5)	3 (5.6)	2 (9.1)	1 (3.1)
Lung and Pleura	30 (6.3)	7 (2.8)	23 (9.9)	30 (7.0)	7 (3.1)	23 (11.5)	0 (0.0)	0 (0.0)	0 (0.0)
Head and Neck	29 (6.0)	8 (3.2)	21 (9.1)	28 (6.6)	8 (3.5)	20 (10.0)	1 (1.9)	0 (0.0)	1 (3.1)
Thyroid	29 (6.0)	17 (6.9)	12 (5.2)	27 (6.3)	16 (7.1)	11 (5.5)	2 (3.7)	1 (4.5)	1 (3.1)
Urinary System	28 (5.8)	8 (3.2)	20 (8.6)	26 (6.11)	7 (3.1)	19 (9.5)	2 (3.7)	1 (4.5)	1 (3.1)
Male Genital System	22 (4.6)	0 (0.0)	22 (9.5)	21 (4.9)	0 (0.0)	21 (10.5)	1 (1.9)	0 (0)	1 (3.1)
Female Genital System	18 (3.8)	18 (7.3)	0 (0.0)	17 (4.0)	17 (7.5)	0 (0.0)	1 (1.9)	1 (4.5)	0 (0)
Brain	17 (3.5)	4 (1.6)	13 (5.6)	10 (2.3)	2 (0.9)	8 (4.0)	8 (14.8)	1 (4.5)	7 (21.9)
Bone and Soft Tissue	15 (3.1)	6 (2.4)	9 (3.9)	9 (2.1)	3 (1.3)	6 (3.0)	5 (9.3)	4 (18.2)	1 (3.1)
Skin	10 (2.1)	3 (1.2)	7 (3.0)	9 (2.1)	3 (1.3)	6 (3.0)	1 (1.9)	0 (0.0)	1 (3.1)
Miscellaneous	8 (1.7)	3 (1.2)	5 (2.2)	5 (1.2)	2 (0.9)	3 (1.5)	3 (5.6)	1 (4.5)	2 (6.3)

**Table 2:** The SEER summary stage at presentation of Palestinian patients with cancer who presented to King Hussein Cancer Center in 2018 and 2019 (N = 480) stratified by age and biological sex.

Stage	Total(n = 480)*n* (%)	Women(n = 200)*n* (%)	Men(n = 226)*n* (%)	Girls and female adolescents(n = 22)*n* (%)	Boys and male adolescents(n = 32)*n* (%)
*In situ*	10 (2.1)	7 (3.1)	3 (1.5)	0 (0.0)	0 (0.0)
Localized	99 (20.6)	44 (19.5)	41 (20.5)	2 (9.1)	12 (37.5)
Regional by direct extension	29 (6.0)	13 (5.8)	14 (7.0)	0 (0.0)	2 (6.3)
Regional to lymph nodes	67 (14.0)	53 (23.5)	12 (6.0)	1 (4.5)	1 (3.1)
Regional both by direct extension and lymph nodes	45 (9.4)	18 (8.0)	26 (13.0)	1 (4.5)	0 (0.0)
Distant	173 (36.0)	59 (26.1)	83 (41.5)	16 (72.7)	15 (46.9)
Unknown	57 (11.9)	32 (14.2)	21 (10.5)	2 (9.1)	2 (6.3)

### 3.3 Clinical characteristics according to age and sex

#### 3.3.1 Adults

Of 426 adult patients, 226 (53.1%) were women and 200 (46.9%) were men. The most common cancer sites in women, in order, were the breast, digestive system, and hematolymphoid system. In men, the most common cancer sites, in order, were the hematolymphoid system, digestive system, and lung and pleura. The mean age at diagnosis was 49 ± 14 years for women and 51 ± 16 years for men. At the time of presentation, 59 women (26.1%) and 83 men (41.5%) had distant metastases.

#### 3.3.2 Children and adolescents

Of 54 children and adolescents, 22 (40.7%) were female and 32 (59.3%) were male. The most common cancer sites in girls and female adolescents were the hematolymphoid system and bone and soft tissue. In boys and male adolescents, the most common cancer sites were the hematolymphoid system and brain. The mean age at diagnosis was 11 ± 6 years for girls and female adolescents and 8 ± 5 years for boys and male adolescents. At the time of presentation, 16 girls and female adolescents (72.7%) and 15 boys and male adolescents (46.9%) had distant metastases.

### 3.4 Clinical characteristics according to the most common cancer sites

#### 3.4.1 Cancers of the breast

The most common cancer site overall was the breast (*n* = 105, 21.9%). The patients were exclusively adults, and all except one were women (*n* = 104, 99.0%). The mean age at diagnosis was 50 ± 13 years. According to the SEER summary stage, the disease was *in situ* in six patients (5.7%), localized in 24 (22.9%), regionalized in 44 (41.9%), distant in 15 (14.3%), and unknown in 16 (15.2%). The predominant type, by histology, was invasive ductal carcinoma (*n* = 101, 96.2%). The remaining cases were invasive lobular carcinoma and mucinous adenocarcinoma (each *n* = 2, 1.9%). Overall, 50 patients (47.6%) underwent surgery, 49 (46.7%) received chemotherapy, 29 (27.6%) received hormone therapy, 17 (16.2%) received radiotherapy, seven (6.7%) received palliative therapy, and four (3.8%) received immunotherapy.

#### 3.4.2 Cancers of the hematolymphoid system

Cancers of the hematolymphoid system were the second most common overall (*n* = 97, 20.2%). The number of adults (*n* = 70, 72.2%) was more than twice the number of children and adolescents (*n* = 27, 27.8%). In addition, the number of male patients (*n* = 63, 64.9%) was almost twice the number of female patients (*n* = 34, 35.1%). The mean age at diagnosis was 32 ± 18 years. According to the SEER summary stage, the disease was localized in six patients (6.2%), regionalized in 12 (12.4%), distant in 72 (74.2%), and unknown in seven (7.2%). The most common type, by histology, was non-Hodgkin lymphoma (*n* = 30, 30.9%), followed by chronic lymphocytic leukemia (*n* = 21, 21.6%), Hodgkin lymphoma (*n* = 19, 19.6%), acute myeloid leukemia (*n* = 9, 9.3%), chronic myeloid leukemia (*n* = 5, 5.2%), multiple myeloma (*n* = 5, 5.2%), and acute lymphocytic leukemia (*n* = 1, 1.0%). Overall, 77 patients (73.3%) received chemotherapy, 20 (19.0%) received palliative therapy, 14 (13.3%) received radiotherapy, 13 (12.4%) underwent transplantation, four (3.8%) underwent surgery, and four (3.8%) received immunotherapy.

#### 3.4.3 Cancers of the digestive system

Cancers of the digestive system were the third most common overall (*n* = 72, 15.0%). Most patients were adults (*n* = 69, 95.8%), and the sexes were equally affected (each *n* = 36, 50.0%). The mean age at diagnosis was 54 ± 17 years. According to the SEER summary stage, the disease was localized in seven patients (9.7%), regionalized in 26 (36.1%), distant in 32 (44.4%), and unknown in seven (9.7%). The most common type was colorectal carcinoma (*n* = 39, 54.2%), followed by gastric carcinoma (*n* = 14, 19.4%), pancreatic adenocarcinoma (*n* = 12, 16.7%), hepatocellular carcinoma (*n* = 4, 5.6%), carcinoid (*n* = 1, 1.4%), cholangiocarcinoma (*n* = 1, 1.4%), and carcinoma of the small intestine (*n* = 1, 1.4%). Overall, 41 patients (56.9%) received chemotherapy, 29 (40.3%) underwent surgery, 21 (29.2%) received palliative therapy, six (8.3%) received radiotherapy, and two (2.8%) received immunotherapy.

## 4 Discussion

We performed the first study of Palestinian patients with cancer seeking care and treatment in Jordan. We described the demographic and clinical characteristics of 480 Palestinian patients treated at KHCC in 2018 and 2019. The most common cancer sites were the breast in female patients and the hematolymphoid system in male patients. Cancers of the digestive system were the second most common in both male and female patients.

Cancer is a leading cause of death in both developed and developing countries (14). Cancer care is challenging even in the best of conditions and becomes exceedingly so in areas of conflict in the Middle East with compromised health care systems (16). Barriers to high-quality cancer care include scarce medical resources, limited access, financial difficulties, absent screening programs, and poor awareness (10, 17).

We found that breast cancer was the most common cancer in Palestinian women treated at KHCC (41.9%). This finding is similar to data from the Palestinian MoH; the rate of breast cancer was 31.3% in the Palestinian territories in 2016 (18). Notably, we also found that 14.3% of Palestinian women with breast cancer treated at KHCC had distant metastasis and 6.7% received palliative therapy. In the Palestinian territories, the 5-year overall survival of women with breast cancer is almost 50% (19). In comparison, the 5-year overall survival of women with breast cancer in the United States, Jordan, and Israel is almost 85% (20, 21). The difference may be attributed to poor breast cancer awareness, lack of screening programs for breast cancer, and limited access to and poor utilization of health care (22). To address the high rate of advanced-stage disease at presentation, we recommend awareness campaigns be held in the Palestinian territories. Awareness campaigns may improve early detection of the most common cancers and lead to better outcomes. Moreover, the implementation of affordable screening programs, especially for cancers of the breast and colorectum, can also improve early detection.

The distribution of cancer sites in our cohort was similar to data from the Palestinian territories for female patients but not male patients. In our study, the most common cancer sites in male patients were the hematolymphoid system, followed by the digestive system and lung and pleura. In the Palestinian territories, the most common cancer sites in men were the lung, followed by the prostate and colorectum (13). The rate of hematolymphoid cancers in our cohort is likely higher because the Palestinian MoH routinely refers patients to KHCC for bone marrow transplantation; only one hospital in the Palestinian territories (namely An-Najah National University Hospital, located in the West Bank) provides bone marrow transplantation and the service is limited (19). It should be noted that the burden of lung cancer among Palestinian may be linked to the Palestinian territories’ lack of a preventive national program for cancer risk. This is best exemplified in the rates of smoking within the Palestinian territories as they are among the highest in the world reaching as high as 26.3% and 56% among the general population and university students, respectively (23). Moreover, Palestinian male students were 10 times more likely to smoke than their female counterparts. Despite the high rates of smoking, the Palestinian MoH did implement WHO tobacco control strategies, raised taxes on tobacco products, and banned their promotion (5). Nonetheless, evidence for the effectiveness of such strategies is yet to emerge.

All patients who present to KHCC undergo baseline diagnostic testing, and all available tissue material and radiographs are reviewed in-center. Interestingly, we found that 7.9% of patients with benign tumors were initially misdiagnosed. Tumors of the central nervous system and soft tissues are often reported by specialists, and pathologists with that expertise are lacking in the Palestinian territories. The shortage of specialists is a significant barrier to optimal cancer care in the Palestinian territories. This barrier may be addressed by virtual multidisciplinary team meetings, twinning between KHCC and hospitals in the Palestinian territories, and other telemedicine solutions. Initiatives to train Palestinian physicians offer numerous advantages. First, the psychosocial outcomes of patients may improve because of the decreased financial burden as well as increased social support. Second, the physical health outcomes of patients may also improve because cancer care is less likely to be interrupted and patients can be followed up for longer durations.

Lack of ancillary testing, which aids in diagnosis and helps distinguish malignant tumors from mimics, may also contribute to misdiagnosis. Many studies have investigated the rate of medical errors in the Palestinian territories, but no specific data are available for misdiagnosis in oncology (24–26). Misdiagnoses may be attributed to the lack of comprehensive cancer care as well as the limited availability of advanced diagnostic equipment. For example, positron emission tomography/computed tomography (PET/CT scan) is not available in the Palestinian territories, there are no nuclear medicine specialists, and patients who require the scan are referred to hospitals in Israel (19). Further studies are required to identify the causes of misdiagnosis, and these causes may be addressed by non-punitive interventions.

The infrastructure of cancer care in the Palestinian territories is deficient and requires investment; there is an urgent need for advanced diagnostic equipment and specialist care. Capacity-building is needed to address the shortage of laboratories, diagnostic equipment, radiation oncology units, bone marrow transplantation units, cancer rehabilitation units, and palliative care wards. For example, with respect to radiation oncology equipment, only two linear accelerators are available to serve the oncologic needs of all Palestinians (1 machine per 1,000,000 inhabitants). These machines are only available in East Jerusalem, thus subjecting patients from Gaza and the West Bank to transportation-related delays (27).

The Palestinian territories lack a comprehensive cancer center that offers specialized, essential cancer care services, such as targeted therapies, multidisciplinary care, palliation, and rehabilitation. Surgery and chemotherapy are generally available, but only one hospital (namely Augusta Victoria Hospital, located in East Jerusalem) offers radiotherapy (19). There are only one radiation oncologist and two medical oncologists per one million population (5). Moreover, to our knowledge, palliative care is generally not available to date in the Palestinian territories. Cancer care is expensive, especially if modalities such as bone marrow transplantation or immunotherapy are indicated. We found that 90.6% of Palestinian patients at KHCC were covered by the Palestinian MoH. However, the MoH only covers the direct costs of Palestinian patients, which presents additional challenges to patients with cancer and their companions, who are not included in the coverage.

Moreover, when analyzing the status of cancer care within the Palestinian territories within a proper geopolitical context, it appears that care defects are not solely oriented around resource scarcity but rather due to asymmetrical racial policies and logistics which originate in the deep historical context of the region (27). Patients with cancer from Gaza and the West Bank may experience significant diagnosis and treatment delays due to the state of inaccessibility set by these policies. These patients are subjected to a myriad of military checkpoints, administrative approvals (e.g., security paperwork and checkups), and permanent roadblocks; all of which may affect the quality of care delivered to and accessed by those patients (28).

We acknowledge two limitations in our study. First, Palestinian patients are followed up for six months following discharge from KHCC, so we could not perform outcome analyses. Second, the Cancer Registry did not restage patients using the 2018 version of the SEER summary staging system if they were initially staged as code 5 using the 2000 version. This largely contributed to missing staging data in 11.9% of patients.

### 4.1 Conclusion

In conclusion, KHCC provides comprehensive cancer care to Palestinian patients referred from the Palestinian MoH. The distribution of cancer sites is similar to data from the Palestinian territories. However, awareness campaigns and screening programs are substandard relative to other countries in the region, which may explain the high rate of advanced-stage disease at presentation. Further studies are required to study the outcomes of Palestinian patients with cancer and the challenges they face during treatment.

## Data availability statement

All data/data sets associated with this project can be requested from the corresponding author at a reasonable request.

## Ethics statement

Studies involving human participants are reviewed and approved by the Institutional Review Board of KHCC. This study was approved under number 20KHCC29. The IRB at KHCC works in accordance with the declaration of Helsinki and its amendments. The IRB granted a waiver of informed consent since the data collected was existing and no patients’ identifiers were collected. Written informed consent from participants or legal guardian/ next of kin was not required in this study in accordance with the national legislation and the institutional requirements.

## Author contributions

Conceptualization: MA-H and AM. Methodology: JZA. Formal analysis: JZA and MA-H. Writing – original draft: RM, and JZA. Writing – review & editing: AA-A, MA-H, and AM. Supervision: MA-H and AM. All authors contributed to the article and approved the submitted version.

## Conflict of interest

The authors declare that the research was conducted in the absence of any commercial or financial relationships that could be construed as a potential conflict of interest.

## Publisher’s note

All claims expressed in this article are solely those of the authors and do not necessarily represent those of their affiliated organizations, or those of the publisher, the editors and the reviewers. Any product that may be evaluated in this article, or claim that may be made by its manufacturer, is not guaranteed or endorsed by the publisher.
